# Supplementary material for: Adverse Childhood Experiences on Reproductive Plans and Adolescent Pregnancy in the Gulf Resilience on Women’s Health Cohort
Source: Int J Environ Res Public Health. 2020 Dec 28;18(1):165. doi: 10.3390/ijerph18010165 (PMC7794759; doi:10.3390/ijerph18010165)
Supplement: Supplementary file 1 [file ijerph-18-00165-s001.pdf]

Supplementary Materials

Table S1. Total and education-stratified crude odds ratios of “Yes” to reproductive plans (*N*=1482).

	Crude Reproductive Plans “Yes” Odds Ratios						
**Exposure**	**Total Sample (*N*=1482)**		**HS or Below (*n*=780)**		**Above HS (*n*=702)**		**Interaction p-value[1]**
Overall ACE	OR	(95 % CI)	OR	(95 % CI)	OR	(95 % CI)	
High	1.67	(1.28-2.19) *	2.40	(1.65-3.49) *	1.08	(0.73-1.60)	<0.01
Low	1.18	(0.87-1.61)	1.59	(1.05-2.4) *	0.81	(0.51-1.29)	0.04
None	1.00	(Ref)	1.00	(Ref)	1.00	(Ref)	
ACE by Age Period	OR	(95 % CI)	OR	(95 % CI)	OR	(95 % CI)	
Childhood							
High	1.79	(1.37-2.33) *	2.49	(1.71-3.63) *	1.22	(0.83-1.80)	0.01
Low	1.30	(0.99-1.70)	1.63	(1.12-2.37) *	0.99	(0.67-1.47)	0.07
None	1.00	(Ref)	1.00	(Ref)	1.00	(Ref)	
Adolescence							
High	1.68	(1.29-2.21) *	2.12	(1.46-3.07) *	1.27	(0.85-1.88)	0.06
Low	1.24	(0.95-1.62)	1.43	(1.00-2.07)	1.01	(0.68-1.51)	0.21
None	1.00	(Ref)	1.00	(Ref)	1.00	(Ref)	
ACE Sub-Type	OR	(95 % CI)	OR	(95 % CI)	OR	(95 % CI)	
Physical Abuse							
High	1.58	(1.20-2.07) *	1.76	(1.20-2.57) *	1.35	(0.92-1.99)	0.34
Low	1.19	(0.92-1.54)	1.75	(1.20-2.54) *	0.80	(0.55-1.16)	<0.01
None	1.00	(Ref)	1.00	(Ref)	1.00	(Ref)	
Emotional Abuse							
High	1.69	(1.32-2.15) *	1.77	(1.26-2.50) *	1.59	(1.12-2.27) *	0.67
Low	1.15	(0.86-1.54)	1.35	(0.91-2.00)	0.96	(0.62-1.47)	0.24
None	1.00	(Ref)	1.00	(Ref)	1.00	(Ref)	
Substance Abuse							
High	1.45	(1.02-2.05) *	1.65	(1.02-2.69) *	1.24	(0.75-2.06)	0.42
Low	1.34	(1.01-1.78) *	1.55	(1.03-2.33) *	1.16	(0.78-1.71)	0.30
None	1.00	(Ref)	1.00	(Ref)	1.00	(Ref)	
Sexual Abuse							
1+ ACE	1.08	(0.82-1.44)	1.29	(0.87-1.91)	0.90	(0.60-1.36)	0.22
None	1.00	(Ref)	1.00	(Ref)	1.00	(Ref)	
Neglect							
1+ ACE	1.15	(0.89-1.48)	1.25	(0.88-1.77)	1.04	(0.72-1.52)	0.49
None	1.00	(Ref)	1.00	(Ref)	1.00	(Ref)	

“Reproductive Plans” was the response to “Looking to the future, do you, yourself, want to have a/another baby at some time?” or “If it were possible, would you, yourself, want to have a/another baby at some time in the future?” “High” exposure ($\geq$3 ACEs) and “Low” ACE exposure (1-2 ACEs) were relative to participants with 0 ACEs (“None”). Five types of ACEs were separately evaluated: sexual abuse, physical abuse without spanking, emotional abuse, neglect, and substance abuse. ACEs experienced during childhood (<12 years old) and adolescence (12-17 years old) were separately examined.

*p<0.05

[1]p-value for statistical interaction between ACE category and educational attainment

Abbreviations: ACE = Adverse Childhood Experience, CI = Confidence Interval, GROWH = Gulf Resilience on Women's Health, HS = High School, OR= Odds Ratio, Ref= Reference.

Table S2. Total and education-stratified crude odds ratios of "Not Sure" to reproductive plans (*N*=1482).

	Crude Reproductive Plans "Not Sure" Odds Ratios					
**Exposure**	**Total Sample (*N*=1482)**		**HS or Below (*n*=780)**		**Above HS (*n*=702)**	
**Overall ACE**	OR	(95 % CI)	OR	(95% CI)	OR	(95% CI)
**High**	1.58	(0.95-2.62)	2.43	(1.19-5.00) *	0.94	(0.46-1.95)
**Low**	0.92	(0.49-1.71)	1.14	(0.47-2.72)	0.70	(0.28-1.71)
**None**	1.00	(Ref)	1.00	(Ref)	1.00	(Ref)
**ACE by Age Period**	OR	(95 % CI)	OR	(95% CI)	OR	(95% CI)
**Childhood**						
**High**	1.89	(1.12-3.18) *	2.26	(1.09-4.69) *	1.50	(0.71-3.17)
**Low**	1.35	(0.79-2.31)	1.70	(0.82-3.52)	1.01	(0.46-2.24)
**None**	1.00	(Ref)	1.00	(Ref)	1.00	(Ref)
**Adolescence**						
**High**	1.51	(0.90-2.53)	2.30	(1.11-4.78) *	0.93	(0.45-1.95)
**Low**	1.21	(0.72-2.02)	1.67	(0.81-3.47)	0.82	(0.39-1.71)
**None**	1.00	(Ref)	1.00	(Ref)	1.00	(Ref)
**ACE Sub-Type**	OR	(95 % CI)	OR	(95% CI)	OR	(95% CI)
**Physical Abuse**						
**High**	1.51	(0.91-2.53)	2.16	(1.09-4.28) *	0.98	(0.45-2.13)
**Low**	1.38	(0.85-2.24)	1.93	(0.96-3.87)	0.97	(0.49-1.92)
**None**	1.00	(Ref)	1.00	(Ref)	1.00	(Ref)
**Emotional Abuse**						
**High**	1.11	(0.68-1.81)	1.39	(0.73-2.65)	0.82	(0.38-1.77)
**Low**	1.33	(0.79-2.22)	1.08	(0.50-2.32)	1.59	(0.78-3.22)
**None**	1.00	(Ref)	1.00	(Ref)	1.00	(Ref)
**Substance Abuse**						
**High**	0.78	(0.34-1.76)	1.23	(0.45-3.35)	0.40	(0.09-1.72)
**Low**	1.66	(1.01-2.70) *	2.21	1.12-4.37	1.22	(0.60-2.48)
**None**	1.00	(Ref)	1.00	(Ref)	1.00	(Ref)
**Sexual Abuse**						
**1+ ACE**	0.43	(0.20-0.90) *	0.33	(0.10-1.09)	0.51	(0.20-1.35)
**None**	1.00	(Ref)	1.00	(Ref)	1.00	(Ref)
**Neglect**						
**1+ ACE**	0.76	(0.44-1.30)	0.91	(0.45-1.84)	0.60	(0.26-1.39)
**None**	1.00	(Ref)	1.00	(Ref)	1.00	(Ref)

Participants responded to "Looking to the future, do you, yourself, want to have a/another baby at some time?" or "If it were possible, would you, yourself, want to have a/another baby at some time in the future?" "High" exposure ($\geq$3 ACEs) and "Low" ACE exposure (1-2 ACEs) were relative to participants with 0 ACEs ("None"). Five types of ACEs and those during childhood (<12 years old) and adolescence (12-17 years old) were separately evaluated.

*p<0.05

Abbreviations: ACE = Adverse Childhood Experience, CI = Confidence Interval, GROWH = Gulf Resilience on Women's Health, HS = High School, OR= Odds Ratio, Ref= Reference..

Table S3. Total crude odds ratios of adolescent pregnancy (*N*=1482).

Exposure	Adolescent Pregnancy	
**Overall ACE**	OR	(95 % CI)
**High**	1.50	(0.94-2.09)
**Low**	1.40	(1.06-2.13) *
**None**	1.00	(Ref)
**ACE by Age Period**	OR	(95 % CI)
**Childhood**		
**High**	1.40	(1.00-1.97)
**Low**	1.31	(0.93-1.85)
**None**	1.00	(Ref)
**Adolescence**		
**High**	1.50	(1.06-2.11) *
**Low**	1.40	(0.99-1.97)
**None**	1.00	(Ref)
**ACE Sub-Type**	OR	(95 % CI)
**Physical Abuse**		
**High**	1.26	(0.91-1.74)
**Low**	1.13	(0.82-1.55)
**None**	1.00	(Ref)
**Emotional Abuse**		
**High**	1.05	(0.78-1.41)
**Low**	0.97	(0.68-1.38)
**None**	1.00	(Ref)
**Substance Abuse**		
**High**	1.60	(1.08-2.38) *
**Low**	0.98	(0.69-1.39)
**None**	1.00	(Ref)
**Sexual Abuse**		
**1+ ACE**	1.65	(1.19-2.29) *
**None**	1.00	(Ref)
**Neglect**		
**1+ ACE**	1.34	(0.99-1.82)
**None**	1.00	(Ref)

"Adolescent Pregnancy" was defined as a reported pregnancy before 18 years old. "High" ACE exposure (≥3 ACEs) and "Low" ACE exposure (1-2 ACEs) were relative to participants with 0 ACEs ("None"). Five types of ACEs were separately evaluated: sexual abuse, physical abuse without spanking, emotional abuse, neglect, and substance abuse. ACEs experienced during childhood (<12 years old) and adolescence (12-17 years old) were also separately examined.

*p<0.05

Abbreviations: ACE = Adverse Childhood Experience, CI = Confidence Interval, GROWH = Gulf Resilience on Women's Health, OR= Odds Ratio, Ref= Reference.
